# The Role of Sustained Attention in the Production of Conjoined Noun Phrases: An Individual Differences Study

**DOI:** 10.1371/journal.pone.0137557

**Published:** 2015-09-03

**Authors:** Suzanne R. Jongman, Antje S. Meyer, Ardi Roelofs

**Affiliations:** 1 Max Planck Institute for Psycholinguistics, Nijmegen, The Netherlands; 2 International Max Planck Research School for Language Sciences, Nijmegen, The Netherlands; 3 Donders Institute for Brain, Cognition and Behaviour, Radboud University, Nijmegen, The Netherlands; Zhejiang Key Laborotory for Research in Assesment of Cognitive Impairments, CHINA

## Abstract

It has previously been shown that language production, performed simultaneously with a nonlinguistic task, involves sustained attention. Sustained attention concerns the ability to maintain alertness over time. Here, we aimed to replicate the previous finding by showing that individuals call upon sustained attention when they plan single noun phrases (e.g., "the carrot") and perform a manual arrow categorization task. In addition, we investigated whether speakers also recruit sustained attention when they produce conjoined noun phrases (e.g., "the carrot and the bucket") describing two pictures, that is, when both the first and second task are linguistic. We found that sustained attention correlated with the proportion of abnormally slow phrase-production responses. Individuals with poor sustained attention displayed a greater number of very slow responses than individuals with better sustained attention. Importantly, this relationship was obtained both for the production of single phrases while performing a nonlinguistic manual task, and the production of noun phrase conjunctions in referring to two spatially separated objects. Inhibition and updating abilities were also measured. These scores did not correlate with our measure of sustained attention, suggesting that sustained attention and executive control are distinct. Overall, the results suggest that planning conjoined noun phrases involves sustained attention, and that language production happens less automatically than has often been assumed.

## Introduction

We talk every day, for years on end. Speaking is such a highly practiced skill that one would think that it must have become a highly automatic process. Yet there are several studies showing that even single word production requires some form of attention [1–3]. Both word production and attention consist of a number of components. Word production involves conceptualizing, lemma retrieval, word-form encoding, and articulation [4], whereas attention includes alertness, orienting, and executive control [5,6], with the latter comprising updating, inhibiting, and shifting [7]. An important question is which production component requires which component of attention. Below, we first briefly describe the major components of word production and attention in more detail. Next, we discuss the scarcity of evidence regarding the attentional demands of the different components of production. The present article concerns the sustained attention demands of phrase production. We report an individual differences study examining the role of sustained attention in the production of complex noun phrases.

Word production consists of several planning stages that a speaker must go through before reaching articulation. There have been several proposals regarding the planning stages [8,9], but here we follow Levelt et al. [4]. Their word production model assumes three planning stages: conceptualizing, lemma retrieval, and word-form encoding, with the word-form encoding incorporating morphological, phonological, and phonetic encoding. First, the concept that best matches the intended message is chosen. The target concept activates the corresponding lemma with its syntactic properties. Then the word form is encoded, which means that the phonological segments of each morpheme are selected and combined into syllables. Finally, the articulatory program is specified (phonetic encoding), which is followed by articulation.

In the present study, we examined how phrase production time depends on sustained attention, which is the ability to maintain alertness over a prolonged period of time [10–12]. Sustained attention research started in the 1940s with Mackworth, who showed radar operators monitoring for rare events tended to increasingly fail to detect such events towards the end of their watch [13]. Since then many studies have shown that people find it hard to stay focused on a task for a long time, even though it is such an essential cognitive capacity, see [10] for a review. Sustained attention is part of the alerting network, one of three anatomically and functionally separate attention networks postulated by Posner and colleagues [5,6,14]. Besides the alerting network, attention consists of orienting and executive control. Orienting denotes the ability to shift the focus of processing to a new spatial source of information, either with eye movement (overtly) or without (covertly). Executive control refers to the ability to remain goal-directed when distracted. Executive control has been decomposed by Miyake and colleagues [7] into updating and monitoring of working memory (updating), inhibiting of prepotent responses (inhibiting), and mental set or task shifting (shifting).

These subcomponents of executive control have previously been linked to language production. Shao, Roelofs, and Meyer [15] showed that individuals with better updating and inhibiting skills were faster at naming pictures than individuals with poorer updating and inhibiting, whereas there was no relationship between shifting ability and word production latency. A correlation between updating ability and picture naming latency was also found by Piai and Roelofs [16].

The orienting of attention during language production has been examined by tracking people's eye gaze (i.e., overt orienting). Saccades and visual attention are tightly coupled, both temporally and spatially [17]. Thus when a person makes a saccade to a new location, his or her visual attention will also be at this new location: Gaze shifts indicate attention shifts. During object naming, people tend to look at the relevant object until they have retrieved the phonological code of the object name, then they shift their gaze towards the next target. For instance, gaze durations are affected by word frequency manipulations [18], phonological priming [19] and word length [20]. These effects are all assumed to occur at the level of phonological encoding. Gaze is held at the target until phonological encoding is complete regardless of whether the next target is linguistic (i.e., another object that needs to be named [21]) or nonlinguistic (i.e., an arrow that needs to be categorized [3]). These findings suggest that orienting of attention is dependent on phonological encoding.

Shifting attention after phonological encoding, but before speech onset, suggests that the final stages of word production, phonetic encoding and the initiation of articulation, can occur in parallel with processes subserving other tasks. This is consistent with the view that late stages in planning a word do not require attention. For instance, Garrod and Pickering [22] argued that the early stages of language production require attention, whereas subsequent processes are automatic. This was corroborated by evidence obtained by Ferreira and Pashler [1], who showed that a semantic manipulation (targeting the early stage of lemma retrieval) influenced performance on a concurrent unrelated task, whereas a phonological manipulation (targeting the later stage of phonological encoding) did not. They concluded that semantic processing could not co-occur with another task, because both tasks tapped into the same central processing resource. In contrast, phonological encoding does not require attention and can therefore proceed in parallel with a second process (but see [2,3]).

However, in a previous study [23] we found evidence that sustained attention does play a role during these last stages of word planning. In this earlier study, we used an individual differences approach to assess the effects of sustained attention ability on early versus late processes of word production. In a first experiment we exploited the finding that gaze durations (i.e., the time from stimulus onset until the overt orienting of attention) reflect the planning processes up to phonological encoding of a word, whereas naming latencies reflect the entire process of word production. Using a dual-task procedure with picture description as Task 1 (e.g., production of the noun phrase "the carrot") and arrow categorization using manual responses as Task 2, we found that naming latencies, but not gaze durations, correlated with sustained attention ability. This suggests that sustained attention is needed for the stages following the gaze shift, namely phonetic encoding and the initiation of articulation. Thus, these final stages do not proceed fully automatically, in contrast to Ferreira and Pashler's [1] conclusion. In a second experiment, we compared picture naming in a dual-task setting (as in the first experiment) to picture naming as the only task. We found significant correlations between naming latencies and sustained attention ability in both tasks. The correlation was, however, significantly stronger in the dual-task than in the single-task setting (*r* = .48 compared to *r* = .35, respectively). Thus, the involvement of sustained attention in naming becomes most evident when sustained attention capacity is shared between picture naming and performing another task.

In the present study we wished to see whether we could increase the need for sustained attention during these final stages of word production without introducing an unrelated, artificial second task. The stronger correlation between naming and sustained attention ability in the dual-task setting in our previous study could have been due to the attention demands of task switching [24–27]. The role of sustained attention could be much diminished when the entire task is linguistic in nature and no task switching is required. This is important to assess, because speakers regularly produce multi-phrase utterances. In the present study we compared the impact of sustained attention on word planning in two dual-task conditions: One task (single object naming hereafter) was identical to the task in the earlier study and required participants to name a picture (e.g., produce the noun phrase "the carrot") and then categorize an arrow as pointing to the left or right. This dual-task situation required switching between a linguistic and a nonlinguistic task. The second task (double object naming) was to name two pictures shown next to each other (e.g., produce the conjoined noun phrase "the carrot and the bucket"). This is also a dual task (with the two naming responses being the two tasks that need to be coordinated), but it does not require switching between a linguistic and a nonlinguistic task. We examined whether strong correlations are observed between sustained attention ability and naming latencies for the first (or only) object name in the switch task only or in both tasks. Should strong correlations be observed only in the switch task we would conclude that sustained attention is only implicated when naming is combined with a nonlinguistic task. However, should strong correlations be observed in both tasks then we would conclude that sustained attention is also involved when participants combine two linguistic tasks, planning two words or phrases in succession, as is required in connected speech.

For each task, half of the blocks contained monosyllabic words and the other blocks consisted of disyllabic words. We expected to replicate Meyer et al. [20], who found a word length effect in pure blocks (i.e., blocks where all words had the same number of syllables) in gaze durations. Finding this effect would allow us to confidently interpret gaze durations as reflecting the processes up to and including phonological encoding. As mentioned previously, in Jongman et al. [23], we observed that sustained attention ability correlated with naming latencies but not with gaze durations. We interpreted this as a late effect of sustained attention, after phonological encoding. Replicating the correlation between sustained attention and naming latencies but not gaze durations, would provide corroborating evidence for our interpretation that sustained attention plays a role especially after phonological encoding, contrary to what is commonly assumed in the literature [1,22].

The literature suggests that although sustained attention and other components of attention are separable, they are also related to some extent. Unsworth, Redick, Lakey, and Young [28] provided evidence that sustained attention ability is related to the updating and inhibiting components of executive control. Moreover, dual-task performance involving picture naming is influenced by individual differences in updating ability [16]. Furthermore, inhibiting ability is engaged in task switching [29], which was required in the dual-task condition of Jongman et al. [23]. To make sure that any correlations between sustained attention ability and language production latency reflected sustained attention rather than updating and inhibiting abilities, we also measured these executive control subcomponents to examine their relationship to sustained attention.

Specifically, we measured updating ability using the operation span task (ospan) and inhibiting ability using the flanker task [7,28]. The operation span task requires participants to solve mathematical equations while having to keep a set of words in working memory. In the flanker task, participants respond to the direction of an arrow, flanked by arrows pointing in the same direction (congruent condition) or in the opposite direction (incongruent condition). Use of the operation span task and the flanker task allowed us to investigate whether sustained attention and these two executive control abilities correlate and therefore whether correlations between sustained attention and language production can be interpreted as purely reflecting sustained attention or also as reflecting executive control. Sustained attention was measured with a digit discrimination task (DDT) [23,30–32]. Sustained attention is typically measured with a continuous performance task, where participants monitor a series of stimuli for a specific, infrequent target (in our experiment the target was presented in 25% of trials). In the DDT, the digit zero is the target amongst the foils zero to nine. Sustaining attention during such a task becomes increasingly difficult due to its repetitive and dull nature.

When investigating the contribution of sustained attention, we did not only look at the mean RTs but also divided the RT distribution into separate components to see whether attention affected a subset of the responses. Using ex-Gaussian analysis one can decompose the underlying RT distribution into two parameters, the μ parameter that reflects the normal part of the distribution and τ which reflects the tail end of the distribution. RT distributions are typically not normally distributed but positively skewed. The τ parameter is an index of skewness. It reflects the proportion of “abnormally” slow responses. Using ex-Gaussian analysis therefore provides much more information than just analyzing the mean RT [33–35].

Jongman et al. [23] found sustained attention ability to correlate only with the τ parameter of the picture description latencies, not μ. In other words, individuals with poorer sustained attention had a greater number of very slow responses when describing a picture compared to individuals with improved sustained attention. We interpreted τ as reflecting lapses of attention as suggested by Unsworth et al. [28]. Similarly, in the object naming study by Shao et al. [15], updating correlated only with the τ parameter of naming latencies, again not with μ. Attention, whether it is alerting or executive control, might be required more for difficult than for easy trials. For the current experiment, we predicted that the correlation between phrase production latency and sustained attention will be found for the τ parameter for both the single and double object conditions.

To summarize, the present study investigated the relationship between sustained attention and the production of conjoined noun phrases as compared to production of a single noun phrase combined with a nonlinguistic manual task. We assessed whether sustained attention ability correlated with the proportion of abnormally slow naming responses for a purely linguistic task (double object naming) and a switch task, or only for the switch task. We expected no correlations between sustained attention and gaze durations in either task, as we hypothesize that the effect of sustained attention is less evident because the early stages of word planning occur without any concurrent competing processing. Finally, we not only measured the participants' sustained attention, but also their updating and inhibiting abilities to test the extent to which the sustained attention task purely reflects sustained attention or also measures executive control.

## Method

### Participants

Sixty-two students of Radboud University Nijmegen or the Hogeschool van Arnhem en Nijmegen took part. All participants were native speakers of Dutch and had normal or corrected-to-normal vision. The average age was 21.4 years (range: 18–28 years) with forty participants being female. Participants were paid for taking part in the study. The current study is part of the approved research program 'Psychology of Language' of Antje Meyer, ethical approval was granted by the Ethics Board of the Faculty of Social Sciences of the Radboud University, Nijmegen. Participants provided written informed consent before the start of the experiment.

### General procedure

Participants first carried out the picture description tasks. The order of the single and double object tasks was counterbalanced across participants. After a break and moving to a different lab, participants performed the flanker task measuring inhibiting ability, then the ospan task measuring updating ability, and finally the digit discrimination task measuring sustained attention ability. The entire session for a single participant lasted one and a half hours.

### Picture description tasks

#### Materials and design

The same materials were used for the single and double object tasks. Participants were presented with 120 black-and-white line drawings selected from a database of normed pictures [36]. Sixty of these pictures had monosyllabic names, the others had disyllabic names. Monosyllabic and disyllabic words were matched for name agreement (which was above 75% for all pictures), frequency, AOA, and visual complexity as measured by file size of the pictures [37]. Initial phoneme was matched pair-wise. Common gender nouns are preceded by the determiner *de*, neuter nouns by *het*. Each set contained the same number of neuter gender nouns (13 out of 60). See S1 File for the set of pictures and S1 Table for their characteristics.

In the double object description task two pictures were presented simultaneously, one in the center of the left half of the computer screen and the other in the center of the right half. Each picture fit into a virtual frame of 5 by 5 cm. In the single object description task, the right picture was replaced by an arrow flanked by xx on each side (font Times New Roman, size 20), yielding xx>xx and xx<xx as stimuli. Each task consisted of four blocks, two blocks containing only monosyllabic words, the other two blocks consisting solely of disyllabic words. Monosyllabic blocks alternated with disyllabic blocks, and the first block was counterbalanced across participants. In the single object task each picture was presented twice, once in each block. In each block of the double object task, each picture was shown twice, once as the initial object, once as the second object. Note that each object was thus named four times, twice in the utterance-initial position and twice in the utterance-final position. For both tasks, pictures were presented in a pseudorandom order, such that participants never named two objects with the same phoneme or from the same semantic category in a row. Each participant had a different order of object presentation.

**Fig 1:**
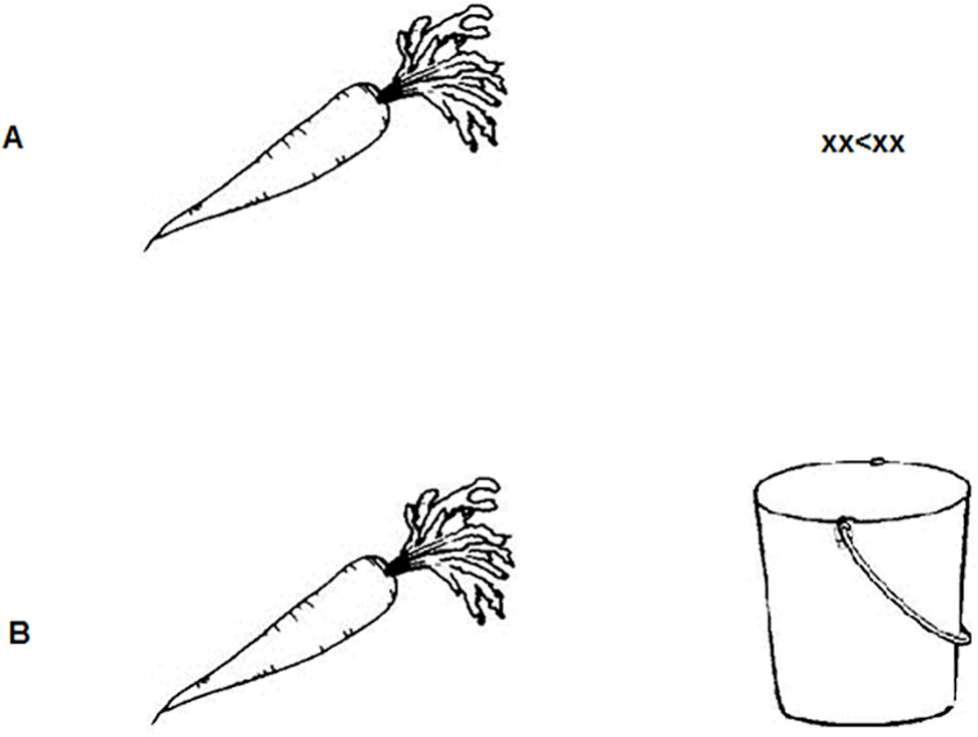
Illustration of the visual stimulus displays used in the single object task (A) and in the double object task (B).

Participants first described the left object and, depending on the task, either indicated the direction of the arrow by a button press or continued by describing the second object (see Fig 1 for an illustration of the displays used). Participants were asked to include the determiner and in the double object task to use the conjunction "en" between the two object descriptions to create conjoined noun phrases such as "de wortel en de emmer" (the carrot and the bucket). Task order was counterbalanced across participants.

#### Procedure

Participants were tested individually in a dimly illuminated room. They were seated in front of a 20 inch screen (Acer TCO03) with their chin on a chin rest, approximately 1 m away from the screen. The movements of each participant's right eye were recorded with an Eyelink 1000 Tower Mount eye tracker sampling at 1000 Hz.

On each trial, a fixation cross was presented for 250 ms in the center of the screen, followed by a blank screen for 150 ms. The stimuli were presented for 3.5 seconds. In the single object task, participants indicated the direction of the arrow by pressing either the left or right arrow on the keyboard (HP KB0316). Spoken utterances were recorded with a Sennheiser ME64 microphone.

#### Analyses

Vocal responses were recorded and RTs were determined manually using the program Praat [38]. Naming errors and hesitations were coded offline and error trials were discarded from the analyses of RTs and gaze durations, as were trials with button press errors. Gaze duration was defined as the time interval between the beginning of the first fixation on the left picture (interest area of 5 by 5 cm) and the end of the last fixation before the first shift of gaze was initiated to the arrow or the second object. The data were analyzed using R [39] and the R packages *lme4* [40] and *languageR* [41]. Gaze durations and naming latencies were both analyzed with a linear mixed effects model with task and word length as fixed effects including their interaction. Fixed effects were centered and the dependent measures were log transformed to eliminate positive skewing. Participant and item were included as random effects [42]. Random slopes were included for all fixed effects to capture additional variability at the subject and item level [43]. The model provides estimates, standard errors and *t*-values for each coefficient; factors with absolute values of *t* greater than 2 were considered to significantly contribute to explaining the dependent variable [44].

### Flanker task

#### Materials and design

The target stimulus, an arrow, was presented in the middle of the screen flanked by two symbols on each side. In the congruent condition, the flankers consisted of arrows pointing in the same direction (e.g., <<<<<) whereas in the incongruent condition the arrows pointed in the opposite direction to the target (e.g., >><>>). In the neutral condition the target was surrounded by xx (e.g., xx<xx). Stimuli were presented in white on a black background (font Arial, size 20) using Presentation Software (Version 16.2, www.neurobs.com). A total of 144 trials were presented separated into two blocks for analysis purposes. Each block contained 24 trials for each of the three conditions, presented in a randomized order.

#### Procedure

Participants were seated in a dimly lit room, facing a 17 inch (Iiyama LM704UT) screen. They were instructed to indicate the direction of the middle arrow as fast and as accurately as possible by pressing either the left or right arrow on the keyboard (HP KB0316). A trial started with a blank screen presented for 1000 ms, this was followed by a fixation cross in the middle of the screen for 250 ms. After another blank screen displayed for 1000 ms, the stimulus was presented. The next trial started after a button press or after 1500 ms. Participants were given a short practice block containing each possible combination of arrow and flankers (6 trials). The task lasted in total approximately 8 minutes.

#### Analyses

RTs were measured and incorrect responses were removed. The correct responses were log transformed to eliminate positive skewing and were analyzed with a linear mixed effects model with condition, block and their interactions as fixed effects. Fixed effects were centered. As condition had three levels, contrast coding was chosen such that the neutral condition was compared to the congruent and incongruent conditions. Participant was included as a random effect. Random slopes for all fixed effects were included.

### Operation span task

#### Materials and design

The equations were taken from Tokowicz, Michael, and Kroll [45]. Sixty mathematical equations were paired with 60 newly chosen Dutch words. All Dutch words were monosyllabic, see S2 File for a list. Each mathematical equation was coupled with a Dutch word (i.e., (15 / 3)- 4 = 1? Pen), presented simultaneously and next to each other in black text on a white screen (font Arial, size 16). The 60 trials were divided into 15 blocks, consisting of 2 to 6 trials. Words within one block differed with respect to their initial phoneme and rhyme. Two practice blocks preceded the experiment. All participants received the same list.

#### Procedure

A trial started with a fixation cross shown in the middle of the screen for 800 ms, followed by a 100 ms blank screen. Then the mathematical equation and word appeared on screen. Participants were requested to first read aloud both the equation and word, then indicate whether the operation was correct or not by pressing either the "Z" or "M" key. After a key press, the next trial started. After a block of trials a recall cue was presented (*herinner "recall"*), and participants were requested to orally recall the words of that block, in the correct order. The experimenter wrote down their responses. The task was self-paced and took on average 15 minutes to complete.

#### Analyses

The operation span score was established using partial-credit unit scoring [46]. For each block, the proportion of correctly recalled words (in the correct serial order) was calculated. Thus correctly recalling one word in a two-word block received the same weight as correctly remembering two words in a four-word block. A participant's ospan score thus reflected the mean proportion of correct items over all blocks and could range from 0 to 1.

### Digit discrimination task

#### Materials and design

Single digits in white (font Arial, size 40) were presented on a black background. The digit 0 was the target digit, and all other digits (1 through 9) were non-targets. Targets were presented with a probability of 25%. Stimuli were presented in a pseudorandom order with the restriction that identical targets never directly followed one another and that targets were preceded by each non-target an equal number of times. A total of 648 trials were presented, divided into a practice block of 72 trials, which was not included in the analysis, and four further blocks of 144 trials (36 targets) each.

#### Procedure

Digits were presented for 100 ms each, with an inter-stimulus-interval of 900 ms. Participants responded to the target stimuli with a button press using their dominant hand. Task duration was 10.8 minutes.

#### Analyses

RTs were measured and errors were divided into misses and false alarms with the former being failures to respond to targets and the latter being responses to non-targets. The RTs for correct responses were log transformed to correct for positive skewing. The linear mixed effects model contained the effect of block (centered) and its random slope, and participant was included as a random effect.

### Analyses of individual differences

For the gaze durations and naming latencies, the ex-Gaussian parameters μ, σ, and τ were estimated using the continuous maximum-likelihood method proposed by Van Zandt [47]. The parameters μ and σ reflect the mean and standard deviation of the normal portion, respectively, and τ reflects the mean and standard deviation of the exponential portion of the distribution. In contrast to the linear mixed effects analyses, latencies were not log-transformed for the ex-Gaussian analyses. The parameters were estimated separately for the single and double object tasks. Moreover, separate analyses were run for monosyllabic and disyllabic words. Therefore, eight sets of parameters (dependent measure by task by syllable length) were estimated for each participant using the program QMPE [48]. We computed Pearson's product-moment coefficients, and tested for correlations between the parameters μ and τ, on the one hand, and the individuals' mean RT and performance decrement (mean RT second half minus mean RT first half) on the DDT, on the other hand. The parameter σ was not included in these analyses because it was not of interest in the present study and to limit the number of comparisons. To test for the relation between sustained attention and executive control, we assessed the correlations between performance on the DDT and individuals' flanker effect and operation span score. To keep the number of correlation tests to a minimum, we did not test for correlations between the flanker effect and ospan scores and the parameters μ and τ of the picture description task. In total, we tested 38 correlations, and applied the Benjamini-Hochberg correction for multiple comparisons. The Benjamini-Hochberg correction controls the false discovery rate instead of the familywise error rate, and as such has more power than Bonferroni-type procedures, especially when the number of tests is large as is the case in the present study [49–52]. The Benjamini-Hochberg procedure first sorts and ranks the *p-*values with the smallest value getting rank 1, the second rank 2 and the largest rank *N*. Then, each *p-*value is multiplied by *N* and divided by its assigned rank. In the present study, this resulted in the first seven correlations to be significant after the Benjamini-Hochberg correction, down to an uncorrected *p*-value of .009.

## Results

Data from four participants were removed. Two participants were excluded because their number of correct math responses in the operation-span task was lower than 80%. This exclusion criterion was used to avoid a trade-off between processing the mathematical equations and storing the words. To allow for ex-Gaussian analyses of the picture description latencies and gaze durations using continuous maximum-likelihood fitting, at least 100 trials per condition are necessary. For one participant, too few eye fixations were recorded due to tracker loss. One participant used the wrong determiner in 1/4th of the naming trials. This left data from 58 participants.

### Picture description tasks

In the single object task, naming errors occurred on 4.5% of the trials. In the double object task, this was true for 8.8% of the trials (4.7% for the left object, 4.3% for the right object). In both tasks, error rates for monosyllabic and disyllabic items were very similar (single: 4.4% and 4.5%; double: 4.5% and 4.5%, respectively). Hesitations occurred on 0.6% of the trials. In the single object task, the wrong arrow direction was chosen on 0.4% of the trials, and on 0.4% of the trials participants indicated the arrow direction before describing the picture, contrary to instructions. All error trials were removed from the following analyses. Moreover, trials with gaze durations to the target below 80 ms or above 2500 ms and trials with production latencies below 400 ms and above 3000 ms were removed, together equating to an additional 0.5% of the data.

The linear mixed effects model for the gaze durations revealed significant main effects for both task (ß = -0.09, SE = 0.03, *t* = -2.68) and word length (ß = 0.06, SE = 0.02, *t* = 3.03). The interaction did not reach significance (ß = 0.01, SE = 0.02, *t* = 0.25). This indicates that gaze durations were significantly shorter for the double object task than for the single object task. Importantly, gaze durations were significantly shorter for monosyllabic words than for disyllabic words. This was true for both tasks, as the interaction between task and word length was not significant (see Table 1).

**Table 1:** Mean latencies per task and per word length for the gaze durations and the vocal responses in the picture description tasks.

		*Gaze*		*Vocal*	
*Task*	*Length*	M	SE	M	SE
Single Object	Monosyllabic	779	4.9	918	3.7
	Disyllabic	828	5.2	947	3.8
Double Object	Monosyllabic	696	4.1	945	3.6
	Disyllabic	739	4.3	964	3.6

M = mean latencies (ms), SE = standard error

The linear mixed effects model for production latencies revealed no significant effects for task (ß = 0.02, SE = 0.02, *t* = 1.08) or word length (ß = 0.02, SE = 0.01, *t* = 1.76). The interaction did not reach significance (ß = 0.01, SE = 0.01, *t* = 1.21).

### Flanker task

On average, 2.7% of the trials were responded to incorrectly. The mean error rates in the incongruent, congruent, and neutral conditions were 7.1%, 0.3%, and 0.7%, respectively. Incorrect responses were removed from the RT analysis. The linear mixed effects model revealed a significant effect of condition. The first contrast, neutral versus congruent condition (445 ms vs. 450 ms), was not significant (ß = 0.01, SE = 0.01, *t* = 1.24). The second contrast, between the neutral and incongruent condition, did show a significant difference (445 ms vs. 556 ms; ß = 0.21, SE = 0.01, *t* = 23.93). Block and the interaction with condition did not reach significance, indicating that performance was stable over time.

### Operation span task

The mean ospan score was 0.59, range 0.20–0.90 (SD = 0.18). The mean score was somewhat lower than reported in other studies using partial-credit unit scoring for the operation span task (M = 0.76, range = 0.54–0.94), although a large range has been shown previously (M = 0.70, range = 0.31–0.92) [16,53].

### Digit discrimination task

Mean RT for the DDT was 408 ms (SE = 0.9). Few errors were made, in total only 0.4% false alarms and 1.3% misses. The linear mixed effects model performed on the RTs showed a significant main effect of block (ß = 0.02, SE = 0.00, *t* = 5.83). As expected, performance speed decreased over time, with an average RT of 392 ms for the first block compared to 424 ms for the final block.

### Analyses of individual differences

Neither of the two executive control measures, the flanker effect or the operation span score, correlated with the mean RTs on the DDT or with the performance decrement on the sustained attention task (flanker: *r* = -.10, *p* = .47 and *r* = .19, *p* = .15; ospan: *r* = -.10, *p* = .47; *r* = -.08, *p* = .54). Mean RTs on the DDT did correlate with individuals' performance decrement on that same task (*r* = .42, *p* = .001), such that participants who were slower in general also showed a larger performance decrement (mean RT second half minus mean RT first half). Note that this correlation remains significant after correcting for multiple comparisons using the Benjamini-Hochberg procedure.

The estimates of the ex-Gaussian parameters of both picture description tasks are presented in Table 2. The correlations between the μ and τ parameters and the two measures of sustained attention (i.e., mean RT and performance decrement on the DDT) are listed in Table 3.

**Table 2:** Mean values of ex-Gaussian parameters per phrase condition for the gaze durations and vocal responses.

		*Gaze*			*Vocal*		
*Task*	*Length*	μ	σ	τ	μ	σ	τ
Single Object	Monosyllabic	563	159	217	692	63	228
	Disyllabic	584	163	248	704	72	247
Double Object	Monosyllabic	493	131	204	731	71	218
	Disyllabic	511	138	226	732	74	235

μ = mu, σ = sigma, τ = tau

**Table 3:** Correlations between ex-Gaussian parameters and sustained attention measures.

			*Single Object*				*Double Object*			
			Monosyllabic		Disyllabic		Monosyllabic		Disyllabic	
*Measure*	*Aspect*		μ	τ	μ	τ	μ	τ	μ	τ
Gaze	DDT	*r*	-.09	.24	-.07	.28	-.15	.42*	-.04	.24
		*p*	.48	.06	.58	.03	.28	.001	.78	.07
	Decr	*r*	-.02	.09	-.08	.29	.04	.18	.04	.22
		*p*	.91	.52	.54	.03	.76	.17	.76	.10
Vocal	DDT	*r*	.20	.46*	.24	.29	.25	.43*	.29	.37*
		*p*	.13	<.001	.08	.03	.06	<.001	.03	.004
	Decr	*r*	.17	.27	.19	.34*	-.01	.31	-.01	.37*
		*p*	.21	.04	.15	.009	.93	.02	.95	.004

DDT = mean latency on the digit discrimination task, Decr = performance decrement on DDT, μ = mu, σ = sigma, τ = tau. Pearson's *r* and uncorrected *p*-values are presented.

*Correlation significant after Benjamini-Hochberg correction for multiple comparisons.

**Fig 2:**
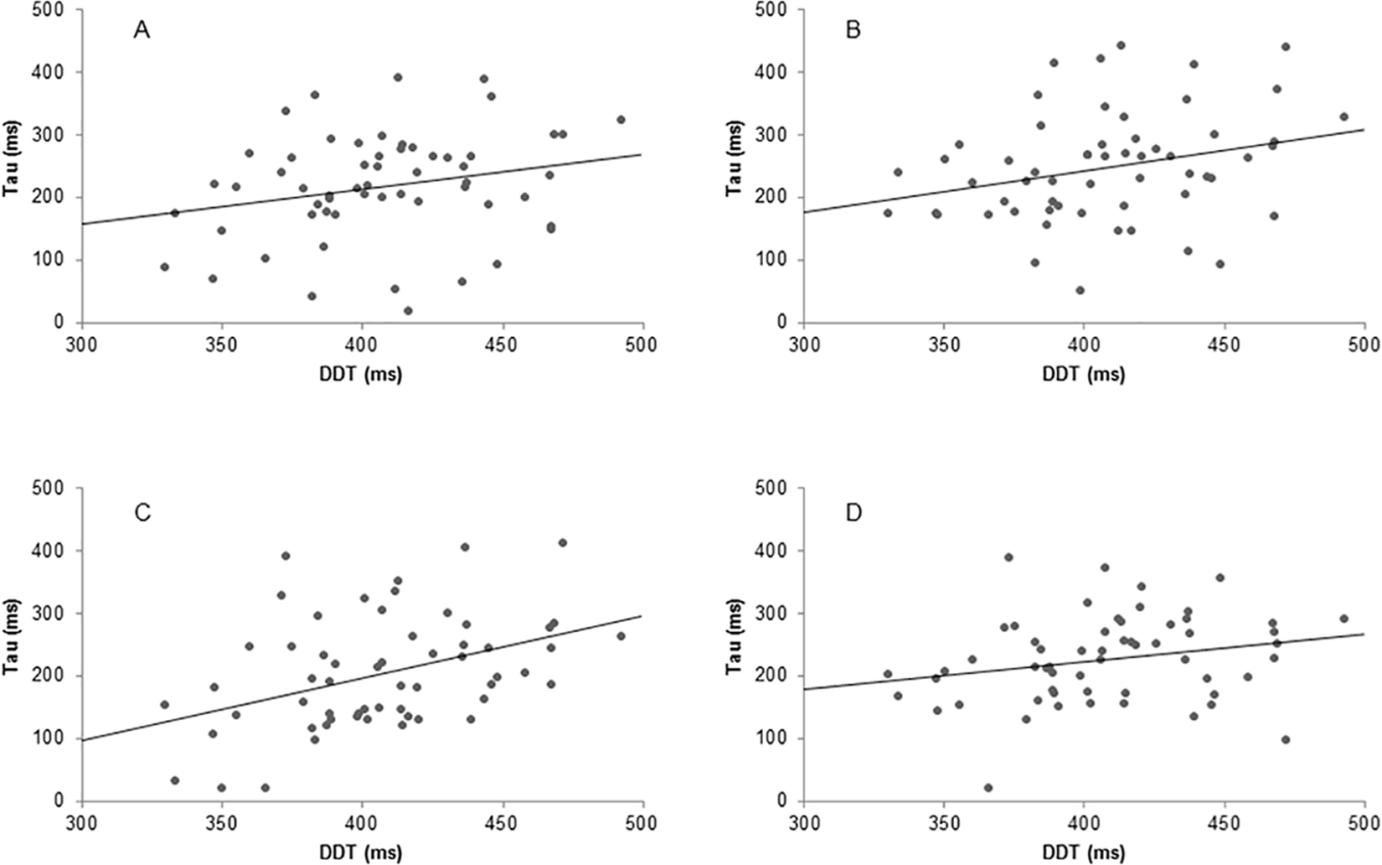
Scatterplots of the relationship between sustained attention and the tau of gaze durations. Tau of gaze durations presented separately for monosyllabic and disyllabic words separately in the single object task (monosyllabic panel A, disyllabic panel B) and the double object task (monosyllabic panel C, disyllabic panel D). Sustained attention is indexed by the mean RT on the digit discrimination task (DDT).

DDT correlated significantly with only one of the four τ parameters estimated for the gaze durations, namely with the monosyllabic words in the double object task: *r* = .42, *p* = .001 (see Fig 2 for all four scatterplots). Individuals' performance decrement on the DDT did not correlate significantly with any of the parameters.

**Fig 3:**
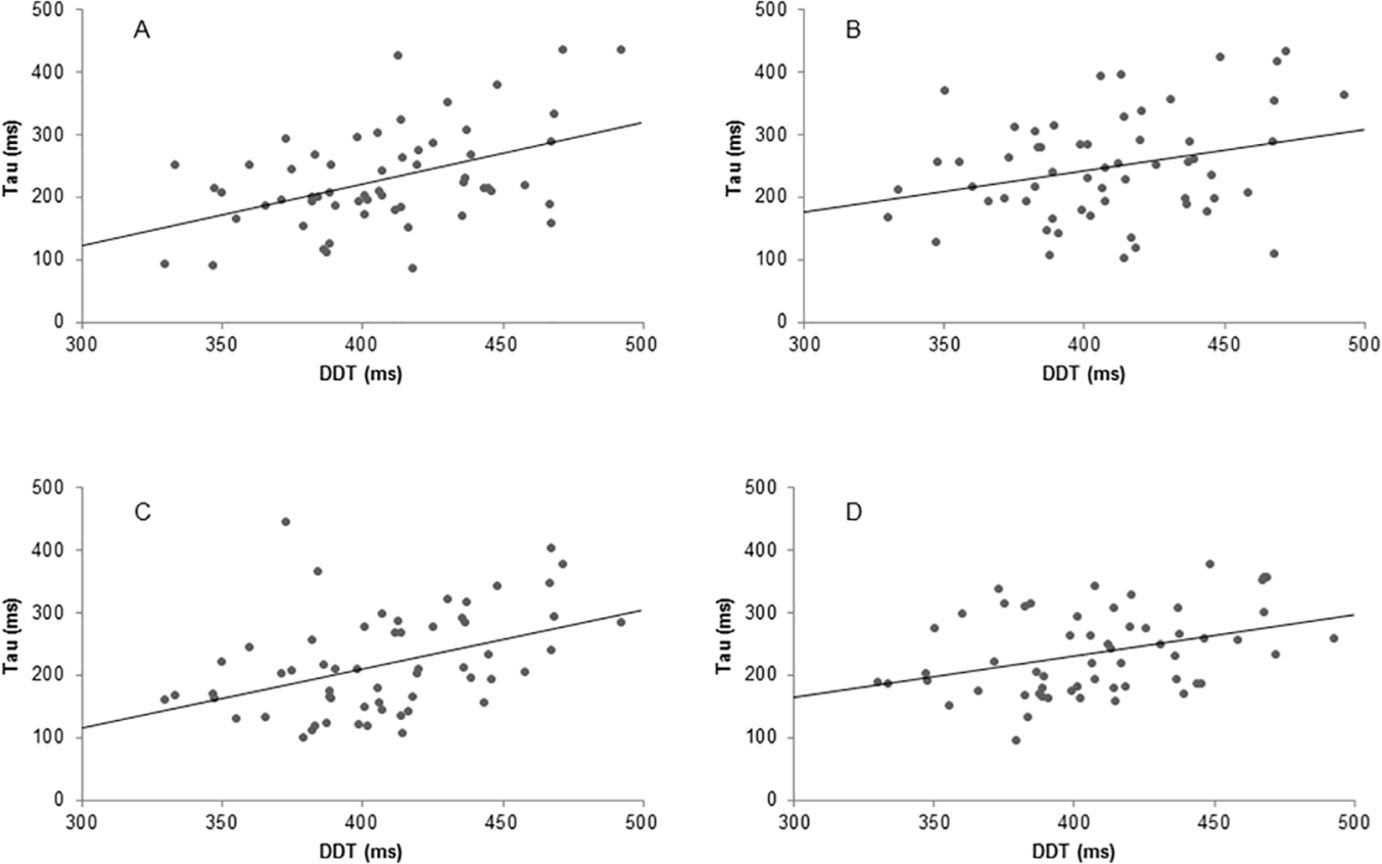
Scatterplots of the relationship between sustained attention and the tau of naming latencies. Tau of naming latencies presented separately for monosyllabic and disyllabic words separately in the single object task (monosyllabic panel A, disyllabic panel B) and the double object task (monosyllabic panel C, disyllabic panel D). Sustained attention is indexed by the mean RT on the digit discrimination task (DDT).

The relationship between DDT and naming latencies was far more stable. Three out of four correlations between the mean RT on the DDT and the τ parameter for the naming latencies were significant after correction for multiple comparisons (see Fig 3 for scatterplots). For the single object task, the monosyllabic word latencies showed a correlation of *r* = .46, *p* <. 001. The double object task showed correlations of *r* = .43, *p* = .001 and *r* = .37, *p* = .004 for monosyllabic and disyllabic words, respectively. Moreover, the τ parameter significantly correlated with the performance decrement for the disyllabic words in both tasks, with correlations of *r* = .34 (single object task) and *r* = .37 (double object task). Thus, individuals with poorer sustained attention, as reflected both by overall slow responding on the DDT and by a larger performance decrement, had a larger number of slow picture description responses independent of task.

The results show a robust effect of DDT on naming latencies and a weaker effect on gaze durations. This is further supported by performing the linear mixed effects model analysis for the picture description data again, this time including the fixed effects flanker effect, operation span score and mean RT on the DDT. For gaze durations, we find again significant main effects of task and word length, with the three attentional measures not contributing to the model. By contrast when we run such a model for production latencies, DDT is the only significant effect (ß = 0.001, SE = 0.00, *t* = 3.16).

## Discussion

The main aim of the current experiment was to test whether speech onset latencies for conjoined noun phrases such as "the carrot and the bucket" correlated with sustained attention ability. We hypothesized that describing two objects in succession would call upon sustained attention because the final processes of producing the first object name coincide with planning the correct phrase for the second object. This hypothesis was derived from results of a previous experiment, where we showed that description latencies for single objects embedded within a dual-task situation correlated with sustained attention ability for the final stages of word production [23]. However, it could be the case that this finding was driven by the switch from a linguistic to a nonlinguistic task. In that case language production in a purely linguistic context might occur without much need for sustained attention. In the present study, we obtained evidence that describing two objects in succession and describing a single object and then carrying out a nonlinguistic task both involved sustained attention to similar degrees.

For both the single and double object naming tasks, we found a correlation between sustained attention ability and the τ parameter of the naming latencies. Individuals with poor sustained attention ability showed a larger number of abnormally slow responses when describing pictures than those with good sustained attention, both when the pictures were followed by another picture to be described and when followed by an arrow categorization task. This suggests that a high level of alertness needs to be maintained to coordinate the production processes of describing an object and the initial processes of a second task regardless of its nature (i.e., linguistic or nonlinguistic). This is corroborated by the finding that performance decrement on the sustained attention task also correlated with production latencies (although after correcting for multiple comparisons this correlation only remained significant for the disyllabic words): Individuals who became increasingly worse in sustaining attention showed an increasing number of slow object description responses compared to individuals who showed no, or a small, performance decrement. Again, this suggests that speakers need to maintain attention when producing complex noun phrases.

The correlation between sustained attention and τ of the naming latencies was consistently present. We also found one out of four correlations (namely with the monosyllabic words in the double object task) to be significant for the mean RTs on the DDT and the τ parameter of gaze durations, contrary to our predictions. In our previous research we found no correlations with gaze durations, only with naming latencies, which we interpreted as an effect of sustained attention on the final processes of word production. Gaze durations have been taken to index the early processes of word planning up to and including phonological encoding [19–21,54]. The effect of sustained attention arose after the gaze shift, which left only phonetic encoding and initiation of articulation to be completed. When this occurred in combination with processing of a second unrelated task, individuals' ability to sustain attention influenced performance. Yet, in the current research we also found a significant correlation with gaze durations suggesting sustained attention is sometimes involved in the early processes of word planning.

The reason why the relationship between sustained attention and the early processes of production becomes evident in the current experiment could potentially be explained by increased task difficulty. A larger picture set was used in this study compared to that used in Jongman et al. [23], with which the participants were not familiarized prior to the experiment, thus making object naming harder. This increased difficulty is reflected in the relatively long RTs. Yet our original conclusion that the relationship between sustained attention and language production becomes increasingly evident when attention needs to be shared with processing of another stimulus receives some support from the current data, as the relationship between sustained attention and the naming latencies was far more stable than for gaze duration. Five out of eight correlations between mean RT and performance decrement on the sustained attention task and the τ parameter of naming latencies reached significance after correcting for multiple comparisons, whereas only one passed the threshold for gaze durations. This suggests that especially the final stages of producing the first object name, phonetic encoding and articulation, are related to sustained attention in complex noun phrase production.

To be certain that indeed only these two processes (i.e., phonetic encoding and initiation of articulation) were left after gaze shifts in our experimental set-up, we included the contrast between monosyllabic and disyllabic words in the current experiment. Meyer et al. [20] showed a word length effect for gaze durations, an effect that takes place at the phonological level. We replicated this result, with gaze durations being longer for disyllabic words than for monosyllabic words. Whether monosyllabic and disyllabic words differ in the amount of sustained attention involved in production cannot be answered by our data. The correlations were smaller for disyllabic words than for monosyllabic words in both the single object task and the double object task, although this difference was only significant for the single object task as calculated by Steiger's *z* (*z* = 2.45, *p* = 0.02). This pattern seems to point to a larger role for sustained attention when producing monosyllabic words. Yet individuals' performance decrement only correlated with the disyllabic words in both tasks, which would support the opposite conclusion that disyllabic words are more tightly linked to sustained attention. Further research is needed to investigate whether certain types of phrases relate differently to sustained attention than others.

In addition to measuring sustained attention, we also used an operation span task and a flanker task to examine the relationship between the updating and inhibiting subcomponents of executive control and sustained attention. Previous research [28] provided evidence that sustained attention ability is related to updating and inhibiting abilities. However, neither updating nor inhibiting ability correlated with sustained attention in our study, which is consistent with the idea that sustained attention is distinct from executive control. This suggests that the observed correlations are instead due to sustained attention rather than being indirect influences of the executive control abilities. However, we must note that our conclusion that sustained attention is needed for language production is based on correlations, and as such we must be careful in interpreting our results. Yet, we favor the simplest explanation where sustained attention is needed for language production instead of a possible third mediating factor that we failed to test in this experiment. If a mediating factor was involved, for instance motivation, we would expect a correlation not just with τ as in the present study, but also with μ as it should affect all trials. To conclude, the present results indicate that sustained attention is involved during the production of conjoined noun phrases. This corroborates and extends earlier evidence that language production happens less automatically than has often been assumed.

## Supporting Information

S1 File Object names picture naming tasks. (DOCX)

S2 File Words operation span task. (DOCX)

S1 Table Characteristics of the object names and pictures used in the picture naming tasks. (DOCX)
