# Supplementary material for: The Role of Sustained Attention in the Production of Conjoined Noun Phrases: An Individual Differences Study
Source: PLoS One. 2015 Sep 3;10(9):e0137557. doi: 10.1371/journal.pone.0137557 (PMC4559420; doi:10.1371/journal.pone.0137557)
Supplement: S1 File — (DOCX) [file pone.0137557.s001.docx]

**File S1. Object names picture naming tasks.**

Monosyllabic words:

arm (arm), aap (monkey), beer (bear), broek (trousers), baard (beard), bril (glasses), bord (plate), bijl (ax), doos (box), dak (roof), eend (duck), geit (goat), harp (harp), hert (deer), jas (coat), kaars (candle), kruis (cross), clown (clown), kok (chef), kers (cherry), knoop (button), leeuw (lion), lamp (lamp), mes (knife), mais (corn), neus (nose), nest (nest), pauw (peacock), pet (hat), peer (pear), pijl (arrow), pijp (pipe), pruik (wig), riem (belt), ring (ring), rits (zipper), roos (rose), rok (skirt), slot (lock), spook (ghost), slak (snail), slee (sled), stuur (steering wheel), spuit (needle), touw (rope), tol (spinning top), tent (tent), tang (pliers), taart (cake), tank (tank), vos (fox), vuur (fire), vlag (flag), vlot (raft), vork (fork), vaas (vase), worst (sausage), wiel (wheel), zaag (saw), zweep (whip).

Disyllabic words:

aardbei (strawberry), anker (anchor), banaan (banana), bureau (desk), beker (trophy), borstel (brush), ballon (balloon), baby (baby), dolfijn (dolphin), dokter (doctor), emmer (bucket), geweer (rifle), handdoek (towel), hamer (hammer), jojo (yoyo), konijn (rabbit), kasteel (castle), cactus (cactus), kanon (cannon), koning (king), ketting (chain), ladder (ladder), lepel (spoon), mixer (mixer), matroos (sailor), neushoorn (rhinoceros), nijlpaard (hippo), pinguin (penguin), pompoen (pumpkin), puzzel (puzzle), potlood (pencil), pleister (bandaid), pizza (pizza), radijs (radish), rugzak (backpack), robot (robot), rolstoel (wheelchair), regen (rain), schildpad (turtle), schouder (shoulder), scharnier (hinge), schommel (swing), springtouw (jumprope), soldaat (soldier), tomaat (tomato), trommel (drum), tafel (table), tandarts (dentist), tractor (tractor), tijger (tiger), varken (pig), vinger (finger), vlieger (kite), vliegtuig (airplane), vleugel (wing), vlinder (butterfly), wortel (carrot), weegschaal (scale), zebra (zebra), zadel (saddle).
