# Supplementary material for: The Role of Sustained Attention in the Production of Conjoined Noun Phrases: An Individual Differences Study
Source: PLoS One. 2015 Sep 3;10(9):e0137557. doi: 10.1371/journal.pone.0137557 (PMC4559420; doi:10.1371/journal.pone.0137557)
Supplement: S1 Table — (DOCX) [file pone.0137557.s003.docx]

**Table S1. Characteristics of the object names and pictures used in the picture naming tasks.**

| Length | Frequency (per million) | Age of Acquisition (yrs) | Name Agreement (%) | Visual Complexity (kb) |
| --- | --- | --- | --- | --- |
| Monosyllabic | 27.3 (SD = 38.1) | 5.8 (SD = 1.2) | 92 (SD = 7.6) | 34.5 (SD = 9.7) |
| Disyllabic | 29.3 (SD = 49.6) | 6.0 (SD = 1.1) | 94 (SD = 6.5) | 36.4 (SD = 11.6) |
